# Hematopoietic Rejuvenation via Natural Senolytic NSPCC1 Delays Inflammatory Aging

**DOI:** 10.3390/biology15120922

**Published:** 2026-06-12

**Authors:** Wei Wang, Shenglong Yang, Rongjinlei Zhang, Yufang Wang, Zhen Zhang, Feng Xiao, Shu Wu, Zhenyu Ju, Ruikun He, Yuanlong Ge

**Affiliations:** 1 State Key Laboratory of Bioactive Molecules and Druggability Assessment, Guangdong Basic Research Center of Excellence for Natural Bioactive Molecules and Discovery of Innovative Drugs, College of Life Science and Technology, Jinan University, Guangzhou 510632, China; 2 Key Laboratory of Regenerative Medicine of Ministry of Education, Institute of Aging and Regenerative Medicine, Department of Developmental & Regenerative Medicine, College of Life Science and Technology, Jinan University, Guangzhou 510632, China; 3 BYHEALTH Institute of Nutrition & Health, Guangzhou 510663, China

**Keywords:** anti-aging, Natural Senolytics, hematopoietic stem cell senescence, inflammatory infiltration, chronic inflammation

## Abstract

Chronic inflammation accelerates the aging process, and targeted clearance of senescent cells shows potential in alleviating age-related decline. PCC1, a potent senescent cell clearance agent in grape seed extract (GSE), has limited applications due to its low oral bioavailability. This study introduced a novel GSE formulation, Natural Senolytics PCC1 (NSPCC1), which significantly enhanced PCC1 absorption and metabolic characteristics. Validation in two mouse aging models demonstrated that oral administration of NSPCC1 markedly extended lifespan and promoted healthy aging. The formulation improved the capacity for hematopoietic stem/progenitor cell differentiation and reduced age-related myeloid cell bias. Comprehensive histological analysis revealed attenuated aging phenotypes in bone marrow and skin, improved peripheral blood erythroid parameters, and a partial increase in blood antioxidant capacity, alongside reduced M1 macrophage infiltration and fibrosis in liver, kidney, and lung tissues. These effects were validated through histological assessments, including H&E, Masson, F4/80, and iNOS staining. This study highlighted the pivotal role of hematopoietic stem cells in aging and established NSPCC1 as a promising natural intervention for age-related pathologies. Its enhanced efficacy lays the groundwork for deeper exploration of natural products in aging biology and provides crucial support for the development of safe and effective anti-aging therapies.

## Simple Summary

Aging is often aggravated by chronic inflammation and the accumulation of senescent cells, which continue to harm surrounding tissues. This study aimed to evaluate whether a new grape seed extract formulation could enhance the delivery of a natural compound called proanthocyanidin C1(PCC1), which could promote healthier aging. The researchers created a formulation named NSPCC1 to improve absorption when taken orally and tested it two aging mouse models. The findings demonstrated that this formulation increased the body’s ability to absorb and utilize PCC1, extended lifespan, and reduced various signs of aging. It improved the function of blood-forming stem cells in the bone marrow, which generate blood and immune cells, and helped restore age-related imbalances in immune cell production. Additionally, it improved blood health, decreased tissue damage, inflammation and immune cell accumulation in organs such as the liver, kidneys and lungs. This research could support the future development of safe, food-based strategies to encourage healthier aging and lessen the impact of age-related diseases.

## 1. Introduction

As one of the core characteristics of the aging process, inflammaging refers to a low-intensity, persistent systemic inflammatory state that develops with advancing age [1,2]. The main mechanism involves elevated levels of pro-inflammatory factors (such as IL-6, TNF-α and CRP) in the bloodstream, creating a chronic inflammatory microenvironment that leads to tissue damage and functional decline [3]. Inflammaging influences the systemic immune system via the bloodstream, creating a positive feedback loop: inflammation promotes the entry of more cells into enter a senescent state, while the accumulation of senescent cells further amplifies inflammation [4]. In other words, inflammatory aging is not only a consequence of aging but can also accelerate it [5]. It is closely associated with various cardiovascular diseases [6]. Therefore, understanding the specific molecular mechanisms by which immune dysregulation drives inflammaging is crucial for advancing translational research.

The decline in immune system function caused by aging of hematopoietic stem cells (HSCs) is closely associated with the onset of age-related diseases and chronic inflammation [7]. HSCs are a population of cells residing in the bone marrow microenvironment, possessing self-renewal capacity and multipotent differentiation potential [8]. As the organism ages, HSCs exhibit reduced self-renewal capacity, limited clonal diversity, and a tendency toward myeloid-biased differentiation [9]. Significant elevation of pro-inflammatory cytokine levels in the bone marrow microenvironment has emerged as a primary trigger for inflammaging [10]. During the aging process, HSCs accumulate substantial endogenous DNA damage and sustained oxidative stress, which strongly activate the NF-κB signaling pathway, further amplifying inflammation [11,12].

Natural products have shown great promise in delaying aging and clearing senescent cells. In recent years, natural compounds such as flavonoids (e.g., apigenin) and stilbene polyphenols (e.g., resveratrol) have been systematically screened and shown to selectively kill senescent cells and improve tissue function [13,14]. PCC1, a natural polyphenolic compound, induces senescent cell apoptosis through multiple pathways, suppresses inflammatory secretion, and achieves senescent cell clearance and lifespan extension across various tissues and models [15]. It represents a significant direction for natural products in anti-aging drug development. However, PCC1’s low oral bioavailability poses a significant challenge to its practical application. Our research has identified a novel grape seed extract, NSPCC1, which exhibits significantly improved bioavailability. Subsequent animal studies have demonstrated that oral administration of NSPCC1 significantly extends the lifespan of old mice. Mechanistically, NSPCC1 modulates the myeloid differentiation potential of HSCs, thereby coordinating the regulation of tissue-specific inflammatory infiltration and conferring systemic anti-aging effects. In conclusion, NSPCC1 shows considerable potential as a natural anti-aging agent.

## 2. Material and Methods

### 2.1. Animal Studies

Sprague Dawley (SD) rats (2–3 months of age), with equal numbers of males and females, were purchased from the Guangdong Medical Laboratory Animal Center to assess the pharmacokinetics of PCC1 following oral administration. The rats were randomly divided into three groups: a PCC1 group (*n* = 8), a GSE group (*n* = 7), and an NSPCC1 group (*n* = 7). Young (2–3 months of age) and old (18–19 months of age) mice of the C57BL/6J were sourced from the Laboratory Animal Management Centre at Jinan University, with an equal number of males and females. After one week of acclimating, the mice were randomly divided into three groups as follows: a Y-Veh (*n* = 10), a O-Veh (*n* = 20), a O-NSPCC1 (*n* = 21). Mice in each group were fed with their corresponding synthetic diets. A standard chow diet and clean water were provided ad libitum. Animals were housed (5 mice per cage) on a 12-h light–dark cycle at 22 ± 2 °C and 55 ± 10% relative humidity.

Sample sizes were determined based on preliminary experiments, prior experience from similar animal studies, the variability of the measured endpoints, and sample availability. Because this study included long-term natural aging observation, survival analysis, histological assessment, flow cytometry, molecular analyses, and in vitro functional assays, sample sizes varied across experiments. Most experiments were not based on a formal prospective power analysis or sample-size calculation.

### 2.2. Chemical Analysis of NSPCC1

This experiment employed a high-resolution liquid chromatography–mass spectrometry system (Orbitrap Exploris 240, Thermo Fisher, Waltham, MA, USA) for compositional analysis of NSPCC1. Sample pretreatment involved thawing, methanol extraction, steel ball grinding, ice bath ultrasonic extraction, centrifugation for phase separation, and low-temperature storage. Samples were resuspended in a 20% methanol/water solution prior to analysis. Data acquisition employed LC-MS analysis methods in positive and negative ion modes. Liquid chromatography was performed on a Thermo Hypersil Gold column (1.9 μm, 2.1 × 100 mm) at a flow rate of 0.30 mL/min. Mass spectrometry acquisition conditions were as follows: a positive ion mode spray voltage of 3.5 kV and a negative ion mode of −2.8 kV, with a full scan resolution of 60,000; MS/MS resolution of 15,000; and a collision energy of 15/30/40 V. After background subtraction of raw data, automatic database searching was performed for qualitative analysis. Detailed results are presented in Table S1.

### 2.3. PCC1 Standard and Synthetic Feeds

The PCC1 standard was purchased from Chengdu Pushi Biotechnology Co., Ltd. (Chengdu, China) (batch number PS011257). The standard was stored at −20 °C under sealed, dry conditions. GSE and NSPCC1 were provided by By-health Co., Ltd. (Guangdong, China) and administered orally at a daily dose of 2 g/kg body weight. The PCC1 standard was dissolved in water to prepare a solution with a concentration of 20 mg/kg. GSE and NSPCC1 were dissolved in water for oral administration to SD rats. The NSPCC1 synthetic diet dosage was 2 g/kg. This 2 g/kg dose was determined based on Sun Yu’s research [15] and a quantitative analysis indicating that the PCC1 content in NSPCC1 was 1% (i.e., the mouse dose was calculated as 20 mg/kg ÷ 1% = 2 g/kg). The vehicle diet complies with the GB 14924.2-2001 hygiene standard [16] and the GB 14924.3-2010 nutritional standard [17] (manufacturer: Guangdong Provincial Center for Medical Laboratory Animals, Guangzhou, China).

### 2.4. TBI

To induce total body irradiation (TBI), male mice aged 2–3 months were exposed to 4.5 Gy of radiation. Following the TBI procedure, mice received either a control diet or an NSPCC1 diet via synthetic feeds for 2 months, after which they underwent further analysis.

### 2.5. Pharmacokinetics in Rats

SD rats (7–8 per group), with equal numbers of males and females, were subjected to a 12-h fasting period prior to drug administration. To comprehensively assess the pharmacokinetics of the administered compound, we established a series of precise sampling time points: prior to administration, and subsequently at 5 min, 15 min, 30 min, 1 h, 2 h, 4 h, 8 h, 12 h, 24 h, and 48 h post-administration. Blood samples were meticulously collected via retro-orbital venous puncture, with each sampling yielding approximately 0.3–0.5 mL of blood per rat. To ensure the integrity of the samples, all blood specimens were anticoagulated with sodium heparin, centrifuged at 2000× *g* for 15 min at 4 °C, and processed within 4 h of collection. The resulting plasma was then carefully aliquoted and stored at −80 °C to maintain its stability for subsequent analyses. Plasma concentrations of PCC1 were measured via LC-MS.

### 2.6. Blood Tests

Blood samples were collected from the tail vein and analyzed for red blood cells (RBC), hemoglobin (HGB), and hematocrit (HCT) levels using the HEMAVET 950FS blood analyzer (Drew Scientific, Miami Lakes, FL, USA).

### 2.7. Measurements of GSH in Serum

To prepare mouse serum samples and prevent post mortem oxidation of glutathione, all assays were completed within two days. The concentrations of reduced glutathione (GSH) and oxidized glutathione (GSSG) were analyzed using a glutathione content detection kit (BC1175, Beijing Solarbio Science & Technology Co., Ltd., Beijing, China). The absorbance of the samples was measured at a wavelength of 412 nm using a microplate reader. The concentration of reduced GSH was determined using the formula [GSH] = [total GSH] − [GSSG] × 2.

### 2.8. Flow Cytometry

Freshly isolated mouse bone marrow cells were washed with PBS, counted, and subjected to flow cytometric analysis. To detect hematopoietic stem and progenitor cells (HSPCs) in bone marrow, cells were first incubated with a lineage marker antibody cocktail at 4 °C for 30 min. The antibody cocktail included anti-CD4 (100508; BioLegend, San Diego, CA, USA), anti-CD8 (BioLegend, 100704), anti-B220 (BioLegend, 103204), anti-CD11b (BioLegend, 101204), anti-Gr-1 (BioLegend, 108404), and anti-TER119 (BioLegend, 116204). After washing, the cells were further incubated with streptavidin-APC-Cy7 (BioLegend, 405208), anti-IL-7R-PerCP-Cy5.5 (BioLegend, 135022), anti-FLT3-PE (BioLegend, 135306), anti-CD150-PE-CF594 (BioLegend, 115936), anti-CD48-BV510 (BioLegend, 103443), anti-Sca-1-PE-Cy7 (BioLegend, 122514), anti-c-Kit-APC (BioLegend, 105812), anti-CD16/32-AF700 (BioLegend, 156620), and anti-CD34-FITC (553733; BD Biosciences, San Jose, CA, USA) at 4 °C in the dark for 2.5 h.

For flow cytometric analysis, cell debris and doublets were first excluded, and gating was performed within the live cell population. Lin^−^Sca-1^+^c-Kit^+^ cells were defined as LSK cells. Within the Lin^−^Sca-1^−^c-Kit^+^ population, common myeloid progenitors (CMPs), granulocyte–macrophage progenitors (GMPs), and megakaryocyte–erythroid progenitors (MEPs) were further identified based on the expression of CD34 and CD16/32 as CD34^+^CD16/32^low^, CD34^+^CD16/32^high^, and CD34^−^CD16/32^low^ cells, respectively. Common lymphoid progenitors (CLPs) were defined as Lin^−^Sca-1^low^c-Kit^low^IL-7R^+^FLT3^+^ cells. HSCs were defined as CD34^−^CD150^+^CD48^−^ cells within the LSK population.

To detect lymphoid and myeloid cells in bone marrow, red blood cells were first lysed with red blood cell lysis buffer (BD Biosciences, 555899) at room temperature for 5 min. After washing, the cells were incubated with anti-CD4-FITC (BioLegend, 100510), anti-CD8-FITC (BioLegend, 100706), anti-B220-APC (BioLegend, 103212), anti-CD11b-PE-Cy7 (BioLegend, 101216), and anti-Gr-1-PE (BioLegend, 108407) at 4 °C in the dark for 30 min. Different gating strategies also use anti-B220-FITC (BioLegend, 103205) and anti-B220-PE-Cy7 (BioLegend, 103221). During analysis, cell debris and doublets were excluded. B220^+^ cells were defined as B cells, CD4^+^ and CD8^+^ cells were used to evaluate the T cell population, CD11b^+^ cells were defined as myeloid cells, and CD11b^+^Gr-1^+^ cells were defined as granulocytes.

Flow cytometric data were acquired on an LSRFortessa flow cytometer (BD Biosciences, Franklin Lakes, NJ, USA) and analyzed using FlowJo software version 10.0.7 R2.

### 2.9. Colony-Forming Unit (CFU) Assay

The colony-forming cell assay, commonly referred to as the methylcellulose assay, is an elegant in vitro technique for quantifying and characterizing hematopoietic progenitor cells [18]. After counting the BM cells, they were plated in MethoCult medium (M3434; STEMCELL Technologies, Vancouver, BC, Canada) according to the manufacturer’s instructions. Briefly, freshly isolated BM cells were cultured at a density of 2 × 10^4^ cells per 35 mm dish for 14 days. The M3434 medium supports optimal growth of erythroid progenitor cells (BFU-E); granulocyte–macrophage progenitor cells (CFU-GM); and multi-potential granulocyte, erythroid, macrophage, and megakaryocyte progenitor cells (CFU-GEMM). The number of colonies was counted under an inverted microscope (Primovert, Carl Zeiss Microscopy GmbH, Jena, Germany).

### 2.10. Bulk RNAseq Analysis

After sequencing was completed on the Illumina high-throughput platform, the raw RNA-seq data were assessed for quality using FastQC (v0.12.1). Adapter sequences and low-quality bases with Q values < 20 were removed using fastp (v1.1.0). High-quality reads were then aligned to the mouse reference genome mm10 using HISAT2 (v2.2.1), and raw gene-level counts were obtained using featureCounts. In the R environment (v4.4.1), the count matrix was normalized to FPKM (fragments per kilobase of transcript per million mapped reads), and principal component analysis (PCA) was performed based on the FPKM values.

Differential expression analysis was performed on the count matrix using DESeq2 (v1.46.0). The Benjamini–Hochberg method was used for multiple testing correction to control the false discovery rate, and the adjusted *p* values were expressed as q values. Genes with |log_2_(fold change)| ≥ 1 and q value < 0.05 were defined as differentially expressed genes (DEGs). Venn diagrams were generated using the VennDiagram package (v1.5.4), and volcano plots were generated using the ggplot2 package (v4.0.1).

Functional enrichment analysis was performed using clusterProfiler (v4.14.6) for Gene Ontology (GO) and Kyoto Encyclopedia of Genes and Genomes (KEGG) pathway enrichment analyses. A *p* value < 0.05 was considered statistically significant. The enrichment degree was evaluated using the enrichment factor, which was calculated as the ratio of the proportion of DEGs assigned to a given pathway to the proportion of background genes assigned to the same pathway.

### 2.11. Histology

Mice were euthanized, and the spleen, skin, liver, kidneys, and lungs were harvested. The tissues were fixed in 4% formaldehyde and embedded in paraffin for histological assessment. Subsequently, 5 µm sections were cut and stained with hematoxylin and eosin (H&E) for histological examination of the respective tissues. Additionally, Masson trichrome staining was performed to evaluate the degree of fibrosis. Representative images were obtained using a scanner and slide viewer software (3D histech, version 2.5.0.143918) for further analysis and evaluation.

### 2.12. Immunohistochemical Analysis

Tissues were fixed in 4% formaldehyde and embedded in paraffin. Then 5 µm sections of the liver, kidneys, and lungs were dewaxed and dehydrated through a series of graded ethanol solutions, followed by antigen retrieval. Next, the sections were treated with 3% hydrogen peroxide in methanol for 15 min to inactivate endogenous peroxidase. After antigen repair, the slides were allowed to cool naturally and washed three times with PBS on a shaker for 5 min each. Subsequently, the sections were blocked with 5% (*v*/*v*) rabbit serum and 3% (*w*/*v*) BSA, then incubated overnight at 4 °C with the primary antibody: F4/80 (rabbit anti-F4/80 polyclonal antibody, 1:1000; servicebio, GB113373) or Inos (rabbit anti-iNOS polyclonal antibody, 1:500; servicebio, GB11119). After washing 3 times with PBS for 5 min each, the sections were allowed to air-dry slightly at room temperature and then stained with an HRP-labeled secondary antibody for 50 min. Following three additional PBS washes, the sections were stained with hematoxylin for 5 min. The sections were then stained with DAB solution, and images were obtained using a scanner and CaseViewer software version 2.5.0.143918 (3DHISTECH Ltd., Budapest, Hungary) for further analysis.

### 2.13. Statistical Analysis

Unless otherwise specified, all data are presented as mean ± SD. Data distribution was first assessed for normality. For comparisons involving more than two groups, the Shapiro–Wilk test was used to assess normality. Data that passed the normality test were analyzed using one-way or two-way ANOVA, followed by Dunnett’s multiple comparison test, as illustrated in the figures. For non-normally distributed data, comparisons between three or more groups were performed using the Kruskal–Wallis test followed by Dunn’s multiple comparisons test. The log-rank (Mantel–Cox) test was employed to compare the survival rates in mice. Statistical differences between groups were analyzed using GraphPad Prism 8.0 software (GraphPad Software, San Diego, CA, USA). The significance levels are indicated as ns (not significant), * *p* < 0.05, ** *p* < 0.01, and *** *p* < 0.001.

## 3. Results

### 3.1. Oral Administration of NSPCC1 Significantly Increased PCC1 Concentrations in Rat Blood

PCC1 was a bioactive polyphenolic compound isolated from GSE. Preclinical evidence indicated that PCC1 exhibited significant senescent cell clearance properties while extending healthspan and lifespan in aged mouse models and premature aging (aging-associated) [19]. However, its limited water solubility and low oral bioavailability necessitate parenteral administration to achieve optimal efficacy, thereby limiting its clinical translation.

**Figure 1:**
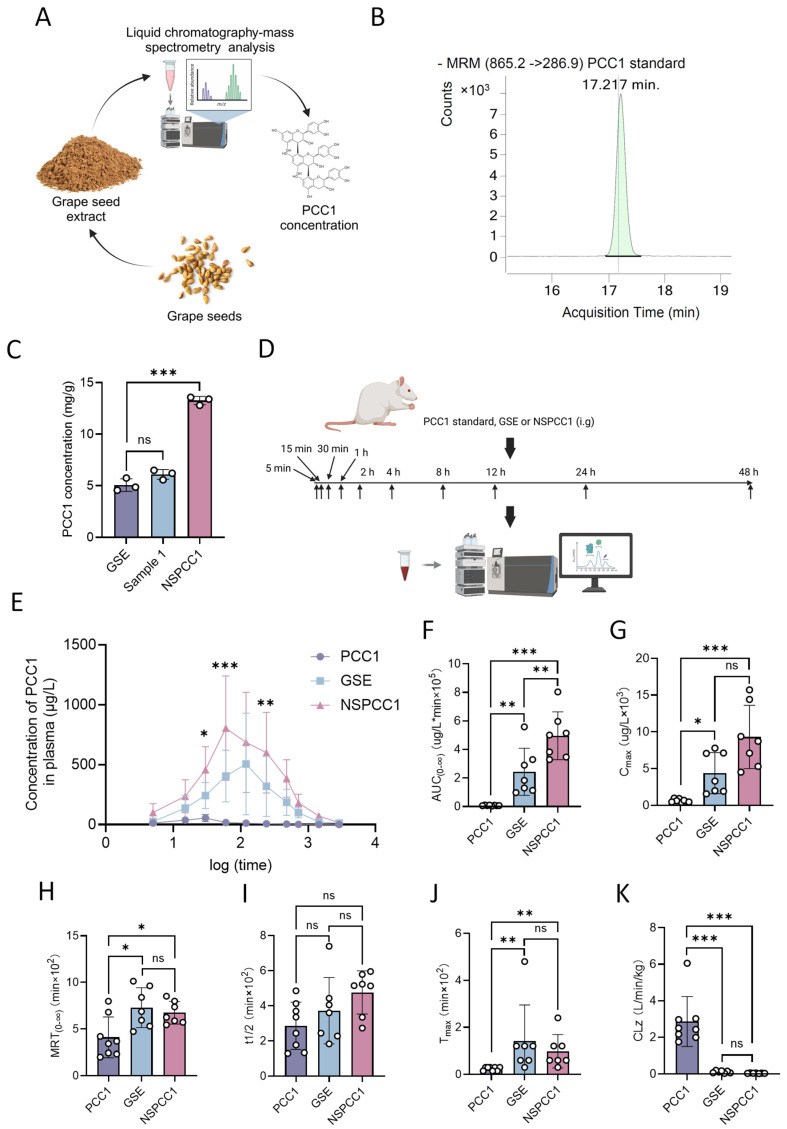
NSPCC1 contained higher PCC1 content and demonstrated better metabolic performance than conventional GSE. (**A**) A schematic illustration of grape seed extract preparation and PCC1 quantification by liquid chromatography–mass spectrometry (LC-MS). (**B**) Representative multiple reaction monitoring (MRM) chromatogram of the PCC1 reference standard. (**C**) The quantification of PCC1 content in conventional GSE, Sample 1, and NSPCC1. (**D**) A schematic diagram of the pharmacokinetic experimental design. Rats were orally administered PCC1 standard, conventional GSE, or NSPCC1, and blood samples were collected at the indicated time points for LC-MS analysis of plasma PCC1 concentration. (**E**) Plasma concentration–time curves of PCC1 after administration of PCC1 standard, conventional GSE, or NSPCC1. (**F**–**K**) Quantitative analysis of pharmacokinetic parameters, including area under the concentration–time curve (AUC), maximum plasma concentration (Cmax), mean residence time (MRT), elimination half-life (t1/2), time to maximum concentration (Tmax), and clearance (CL). These parameters were used to evaluate the absorption, systemic exposure, residence time, and elimination characteristics of PCC1 among the different formulations. Data are presented as mean ± SD. For PCC1 content analysis, each dot represents an independent sample. For pharmacokinetic analysis, *n* = 7–8 rats per group. Statistical significance among the three groups was analyzed using one-way ANOVA, followed by Tukey’s multiple comparisons test. For datasets that did not pass the Shapiro–Wilk normality test, the Kruskal–Wallis test followed by Dunn’s multiple comparisons test was used. Nonparametric tests were applied to Cmax, Tmax, and CL. ns, not significant; * *p* < 0.05, ** *p* < 0.01, *** *p* < 0.001.

To screen GSE enriched with high-concentration PCC1, we employed conventional extraction methods and innovative processing techniques to produce distinct GSE formulations. The PCC1 content in each extract was quantified via liquid chromatography-mass spectrometry (LC-MS) analysis (Figure 1A). Our findings indicated that NSPCC1, a novel natural senolytic PCC1 formulation, exhibited the highest PCC1 concentration compared with the conventional GSE and other innovative extraction (sample 1) (Figure 1B,C). Consequently, NSPCC1 was selected for further investigation in aging assessments, and its major components were analyzed via mass spectrometry (Table S1). Pharmacokinetic evaluations were performed on rats administered 20 mg/kg PCC1 of standard, 2 g/kg of GSE, or 2 g/kg of NSPCC1. Blood samples were collected from each group for PCC1 quantification analysis (Figure 1D). The pharmacokinetic analysis revealed that systemic PCC1 exposure was significantly increased after NSPCC1 administration compared with traditional GSE, whereas absorption of the oral PCC1 reference standard was negligible (Figure 1E).

Additionally, pharmacokinetic analysis showed that the area under the curve (AUC) was significantly higher in the NSPCC1-treated group than in the GSE-treated group (Figure 1F), whereas the maximum plasma concentration (Cmax) showed an increasing trend but did not differ significantly between these two groups (Figure 1G). Notably, the time to maximum concentration (Tmax) for the PCC1 standard, GSE, and NSPCC1 remained consistent at around 2 h, indicating rapid absorption kinetics across all treatment groups (Figure 1J). These results demonstrated that NSPCC1 provides superior systemic exposure to PCC1 compared with traditional GSE, while maintaining comparable absorption kinetics, clearance rates, and duration (Figure 1F–K). Consequently, NSPCC1 served as a more efficient vehicle for PCC1 delivery than conventional GSE, without altering its fundamental pharmacokinetic behavior.

### 3.2. NSPCC1 Maintained Physiological Function and Extends Lifespan in Aged Mice

**Figure 2:**
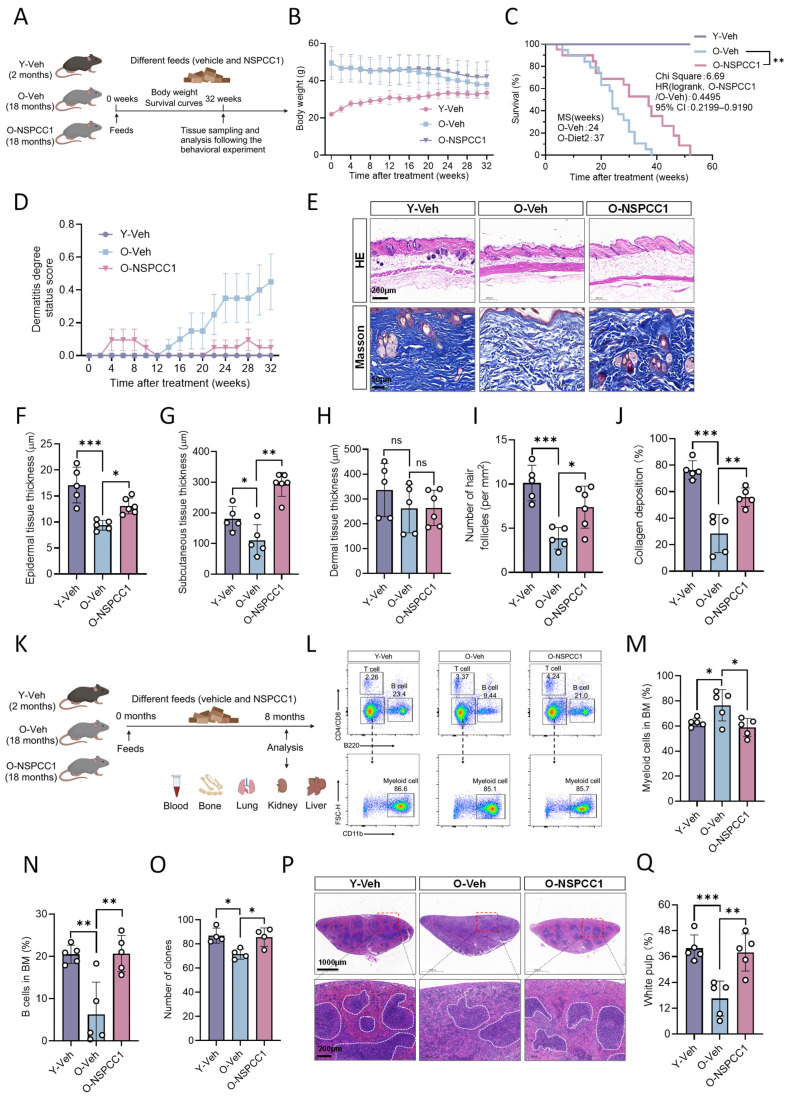
NSPCC1 significantly delayed aging. (**A**) A schematic illustration of the experimental design. Y-Veh (2 months old), O-Veh (18 months old), and O-NSPCC1 (18 months old) were subjected to dietary intervention. Body weight, survival, and dermatitis status were monitored during the 32-week treatment period, followed by tissue collection and behavioral analysis. (**B**) Longitudinal analysis of body weight during the treatment period. (**C**) Kaplan–Meier survival curves of mice in the indicated groups. The median survival time was calculated for each group. (**D**) Longitudinal analysis of dermatitis severity scores during the treatment period. Dermatitis status was evaluated at the specified time points using predefined scoring criteria. (**E**) Representative H&E and Masson’s trichrome staining images of skin tissues after 32 weeks of dietary intervention. (**F**–**J**) Quantitative histomorphometric analysis of skin tissues, including epidermal thickness (**F**), subcutaneous adipose tissue thickness (**G**), dermal thickness (**H**), hair follicle density (**I**), and collagen deposition (**J**). Collagen deposition was quantified as the percentage of Masson-positive area. (**K**) A schematic illustration of tissue collection after 8 months of dietary intervention. Blood, bone marrow, spleen, lung, kidney, and liver samples were harvested from Y-Veh, O-Veh, and O-NSPCC1 mice for subsequent analyses. (**L**) Representative flow cytometry images of bone marrow cell populations. Single-cell populations were analyzed after excluding debris and duplexes. T cells were defined as CD4^+^ and CD8^+^ cells, B cells as B220^+^ cells, and myeloid cells as CD11b^+^ cells. (**M**,**N**) Quantitative analysis of myeloid cells (**M**) and B cells (**N**) in the bone marrow. (**O**) Quantitative analysis of total colony numbers in bone marrow colony-forming assays after 10 days of in vitro culture, reflecting the clonogenic capacity of hematopoietic progenitor cells. (**P**) Representative H&E-stained spleen sections showing the structure of splenic white pulp in the indicated groups. (**Q**) Quantitative analysis of splenic white pulp area. For panels (**A**–**D**), sample sizes were as follows: Y-Veh, *n* = 10; O-Veh, *n* = 20; and O-NSPCC1, *n* = 21. For skin histological analysis, *n* = 5–6 mice per group. For bone marrow flow cytometry and spleen histological analysis, *n* = 5 mice per group. For colony-forming assays, *n* = 4 biological replicates per group. Data are presented as mean ± SEM. Survival curves were analyzed using Kaplan–Meier survival analysis, followed by the log-rank Mantel–Cox test. ns, not significant; * *p* < 0.05, ** *p* < 0.01, *** *p* < 0.001.

To evaluate the anti-aging potential of NSPCC1, we fed 18-month-old mice either a vehicle feed or an NSPCC1-enriched synthetic feed. Longitudinal monitoring included body weight measurements, surface changes, and survival curve analysis (Figure 2A). Although the treatment had no significant effect on body weight (Figure 2B), it extended median survival by at least 10% (Figure 2C), and no significant sex differences were observed (Figure S1A,B). Notably, mice on the vehicle diet exhibited significant skin inflammation and lesions after 32 weeks of dietary intervention, whereas NSPCC1 supplementation substantially ameliorated these lesions (Figure 2D). Consistent with established aging phenotypes, hematoxylin and eosin (HE) staining revealed altered skin architecture, characterized by reduced tissue thickness and decreased collagen deposition (Figure 2E). Quantitative analysis demonstrated that NSPCC1 administration significantly improved epidermal and subcutaneous tissue thickness (Figure 2F,G). Although no effect on dermal tissue thickness was observed (Figure 2H), there was a significant increase in hair follicle density (Figure 2I) and a marked rise in collagen content (Figure 2J), suggesting that NSPCC1 had the potential to modulate extracellular matrix composition.

After 32 weeks of dietary intervention, behavioral assessments were conducted (Figure S1C). Results indicated that NSPCC1 treatment did not affect the total moved distance (open field test), average speed (open field test), center frequency (open field test) (Figure S1D,F), recognition index (novel object recognition test) (Figure S1G), and rotarod duration (Rotarod test) (Figure S1H). However, NSPCC1 significantly suppressed the decline in limb grip strength (grip meter) in aged mice (Figure S1I). These results demonstrated that although NSPCC1 failed to alleviate age-related neurological deficits, neither cognitive nor motor coordination parameters were affected; it did exhibit a protective effect on muscle strength.

To investigate the effects of NSPCC1 on the hematopoietic system, 18-month-old mice were fed either a vehicle diet or an NSPCC1 diet for 8 months, while 2-month-old mice were used as the Y-Veh group. At the end of the intervention, peripheral blood, bone marrow, and spleen samples were collected for further analysis (Figure 2K). Previous studies have shown that hematopoietic aging is characterized by a decline in hematopoietic stem/progenitor cell function, an increase in myeloid cells, and a reduction in lymphoid cells during natural aging [20]. Therefore, we first examined the proportions of T cells, B cells, and myeloid cells in the bone marrow, and further evaluated the in vitro colony-forming capacity of bone marrow hematopoietic stem/progenitor cells. The results showed that, compared with the Y-Veh group, the O-Veh group exhibited an increased proportion of myeloid cells and a decreased proportion of B cells in the bone marrow. After NSPCC1 intervention, the proportion of myeloid cells was significantly reduced (Figure 2L,M), whereas the proportion of B cells was significantly increased (Figure 2L,N). In contrast, no significant change was observed in the proportion of T cells (Figure S2A). Furthermore, the in vitro colony-forming assay showed that bone marrow cells from the NSPCC1-treated group formed more total colonies (Figure 2O), and this increase was mainly attributable to enhanced CFU-GM formation (Figure S2B–D). Together, these results suggested that NSPCC1 partially ameliorated aging-associated bone marrow lineage imbalance and enhanced the in vitro hematopoietic capacity of bone marrow progenitor cells.

The spleen plays multiple roles in the hematopoietic system, including hematopoiesis, blood filtration, immune defense, blood storage, and regulation of iron metabolism [21], thereby establishing its critical function in maintaining blood homeostasis and overall immune function [22]. In aged mice, dual atrophy of the red and white pulp was observed, along with reduced white pulp cell density and extramedullary hematopoiesis [23]. Histopathological examination using H&E staining revealed significant degeneration of the white pulp in the O-Veh group, whereas NSPCC1 administration exerted a protective effect, markedly attenuating these pathological alterations (Figure 2P,Q).

Further comparative analysis of peripheral blood parameters across mouse groups revealed that NSPCC1 treatment improved peripheral erythrocyte hematological parameters in aged mice compared with naturally aging mice (Figure S2E). Specifically, NSPCC1 supplementation significantly mitigated age-associated declines in RBC count, HGB concentration, and HCT levels (Figure S2F–H). Notably, total glutathione (T-GSH) and GSH concentrations in peripheral blood were significantly elevated in the NSPCC1 group, indicating enhanced antioxidative capacity (Figure S2I,J).

These findings suggested that NSPCC1 might enhance hematopoietic function and peripheral blood parameters by regulating hematopoietic stem and progenitor cells (HSPCs) and their downstream differentiation pathways.

### 3.3. NSPCC1 Attenuated Fibrosis in Tissues and Organs of Naturally Aged Mice

**Figure 3:**
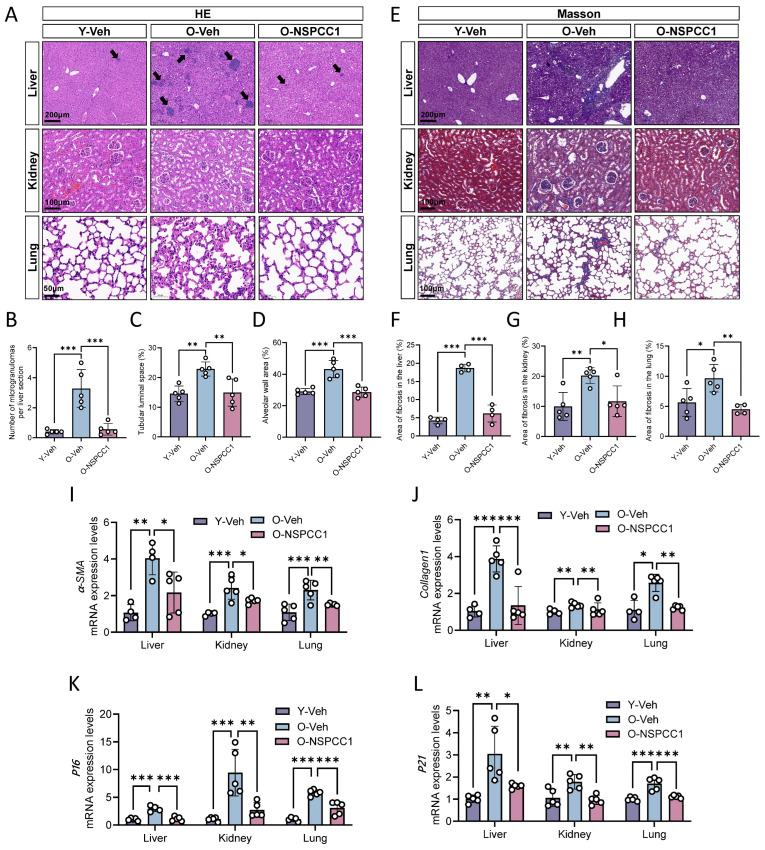
NSPCC1 significantly ameliorated tissue fibrosis across multiple organs in naturally aged mice. (**A**) Representative H&E-stained images of liver, kidney, and lung tissues from Y-Veh, O-Veh, and O-NSPCC1 mice. Arrows indicate hepatic microgranulomas. Scale bars: 200 μm for the liver, 100 μm for the kidney, and 50 μm for the lung. (**B**–**D**) Quantitative histopathological analysis of liver, kidney, and lung tissues, including the number of hepatic microgranulomas (**B**), the percentage of tubular lumen area in the renal cortex (**C**), and the percentage of damaged area in lung tissue (**D**). (**E**) Representative Masson’s trichrome staining images of liver, kidney, and lung tissues from the indicated groups. Collagen deposition is shown in blue. Scale bars: 200 μm for liver and 100 μm for the kidney and lung. (**F**–**H**) Quantitative analysis of fibrotic area in the liver (**F**), kidney (**G**), and lung (**H**). The fibrotic area was quantified as the percentage of Masson-positive collagen area relative to the total tissue area. (**I**,**J**) qPCR analysis of fibrosis-related genes, including α-smooth muscle actin (α-SMA) (**I**) and collagen type I (Collagen 1) (**J**), in liver, kidney, and lung tissues. (**K**,**L**) qPCR analysis of senescence-related genes, including p16 (**K**) and p21 (**L**), in liver, kidney, and lung tissues. Data are presented as the mean ± SD. Each dot represents one mouse or one biological replicate. Histological quantification was performed using ImageJ software version 1.53f from representative fields of each tissue section. Differences between the three groups were analyzed using one-way ANOVA followed by Tukey’s multiple comparisons test for normally distributed data. ns, not significant; * *p* < 0.05, ** *p* < 0.01, *** *p* < 0.001.

Aging was accompanied by distinctive changes in the structure and morphology of various tissues and organs. To determine these changes, we performed histopathological evaluation of the liver, kidney, and lung tissues from each group of mice. Previous studies have indicated that the aging process induces characteristic age-related hepatic alterations, including fat accumulation, fibrosis, inflammatory/immune dysregulation, and cancer [24,25]. Our histopathological analysis of liver-specific aging phenotypes revealed a significant increase in microgranuloma formation in aged mice, whereas the NSPCC1 treatment group exhibited markedly reduced microgranuloma numbers (Figure 3A,B). In renal tissues, age-associated pathological changes manifested as glomerulosclerosis, loss of renal parenchymal mass, and hyalinosis of afferent arterioles [25,26,27]. Notably, aged kidneys exhibited marked tubular epithelial atrophy accompanied by luminal dilation [28]. Quantitative assessment of tubular dimensions via H&E-stained renal sections revealed significant age-dependent enlargement, which was markedly improved by NSPCC1 administration (Figure 3C). During aging, lung tissues exhibited progressive thickening of alveolar walls, attributable to excessive deposition of collagen and other extracellular matrix components [1]. Notably, NSPCC1 administration significantly alleviated these structural abnormalities, with histopathological examination revealing markedly reduced alveolar wall thickening (Figure 3D).

Masson staining revealed significant collagen deposition in the livers, kidneys, and lungs of aged mice. Following treatment with NSPCC1, this structural change was significantly reduced (Figure 3E–H). Quantitative PCR (qPCR) data further demonstrated a significant decrease in mRNA expression levels of fibrosis markers α-SMA and Collagen 1 after treatment (Figure 3I,J).

To further evaluate senescence-associated molecular changes in naturally aged mice, we examined the expression of p16 and p21 in the liver, kidney, and lung. Compared with the young control group, aged mice exhibited elevated expression of p16 and p21 in these tissues. NSPCC1 treatment reduced the expression of both markers, although the magnitude of change varied across organs (Figure 3K,L). These findings suggested that NSPCC1 was associated with attenuation of senescence-related molecular features in naturally aged tissues.

These findings indicated that NSPCC1 can alleviate fibrotic changes in naturally aging mice and reduce the expression of senescent cell-related biomarkers.

### 3.4. NSPCC1 Delayed Aging by Reducing Inflammatory Infiltration in Tissues of Aged Mice

**Figure 4:**
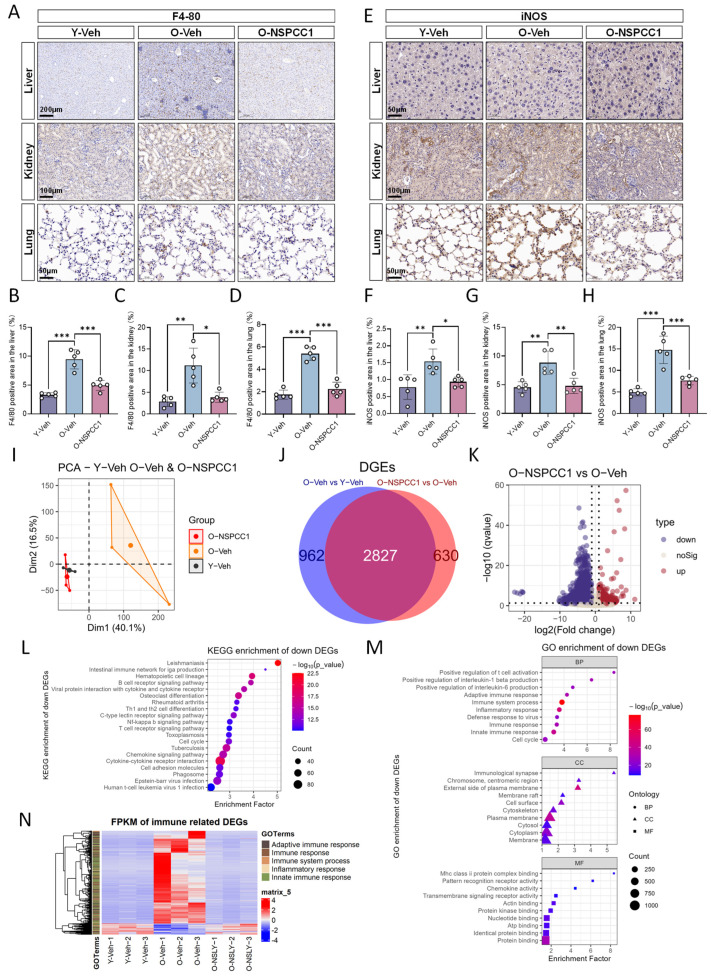
NSPCC1 mitigated inflammatory response and macrophage infiltration to delay aging in natural aging models. (**A**) Representative immunohistochemical staining images of F4/80 in liver, kidney, and lung tissues from Y-Veh, O-Veh, and O-NSPCC1 mice. F4/80 was used as a macrophage marker. Scale bars: 200 μm for liver, 100 μm for kidney, and 50 μm for lung. (**B**–**D**) Quantitative analysis of F4/80-positive staining area in the liver (**B**), kidney (**C**), and lung (**D**). The F4/80-positive area was quantified as the percentage of positive staining area relative to the total analyzed tissue area. (**E**) Representative immunohistochemical staining images of iNOS in liver, kidney, and lung tissues from the indicated groups. iNOS was used as a marker of pro-inflammatory M1-like macrophages. Scale bars: 50 μm for the liver, 100 μm for the kidney, and 50 μm for the lung. (**F**–**H**) Quantitative analysis of iNOS-positive staining area in the liver (**F**), kidney (**G**), and lung (**H**). The iNOS-positive area was quantified as the percentage of positive staining area relative to the total analyzed tissue area. (**I**) PCA of hepatic transcriptomes from Y-Veh, O-Veh, and O-NSPCC1 groups (*n* = 3 biologically independent samples). (**J**) Venn diagram depicting overlapping DEGs between Y-Veh vs. O-Veh (age-related changes) and O-NSPCC1 vs. O-Veh (treatment effects). (**K**) Volcano plot of DEGs (|log2FC| > 1, Q value < 0.05) comparing O-NSPCC1 and O-Veh groups. (**L**–**N**) Functional analysis of downregulated DEGs: (**L**) KEGG pathways, (**M**) GO terms, and (**N**) heatmap of the expression patterns of DEGs associated with enriched immune/inflammation-related terms; data are shown as FPKM. For immunohistochemical quantification, data are presented as mean ± SD, and each dot represents one mouse or one biological replicate. For transcriptomic analysis, *n* = 3 biologically independent liver samples were used per group. Differences between the three groups were analyzed using one-way ANOVA followed by Tukey’s multiple comparisons test for normally distributed data. For datasets that did not pass the Shapiro–Wilk normality test, the Kruskal–Wallis test followed by Dunn’s multiple comparisons test was used. Nonparametric analysis was applied to the F4/80-positive area in the kidney. ns, not significant; * *p* < 0.05, ** *p* < 0.01, *** *p* < 0.001.

During aging, senescent cells accumulated prominently, causing tissue dysfunction. They secreted various cytokines/chemokines to recruit immune cells for clearance, but the senescent microenvironment induced immunosenescence, reducing clearance efficiency [29]. Residual senescent cells persistently activated the CCL2/CCR2 axis, recruiting monocytes that differentiated into pro-inflammatory M1 macrophages, which secreted TNF-α/IL-1β, thereby fueling chronic inflammation. Simultaneously, overactivated TGF-β/Smad3 signaling activates fibroblasts, creating a profibrotic environment. Thus, immune cells acted as critical regulators in fibrotic progression [30]. To evaluate the immunomodulatory capacity of NSPCC1, we performed immunohistochemical staining for F4/80 (pan-macrophage marker) and iNOS (M1 macrophage marker) in mouse liver, kidney, and lung tissues. Quantitative analysis revealed that NSPCC1 treatment significantly reduced macrophage infiltration (Figure 4A–D), and decreased the number of M1 macrophages (Figure 4E–H).

Then, we performed RNA sequencing with the liver tissue, which exhibited the most pronounced aging phenotype. PCA revealed significant separation between the O-NSPCC1 group and O-Veh group, with the transcription patterns of the O-NSPCC1 group more consistent with those of the Y-Veh group (Figure 4I). Comparative analysis of DEGs between O-Veh vs. Y-Veh and O-NSPCC1 vs. O-Veh groups revealed that the majority of DEGs were consistent, indicating that the genes regulated by NSPCC1 were functionally associated with aging (Figure 4J).

Analysis of differentially expressed genes identified 3060 downregulated genes and 1399 upregulated genes in O-NSPCC1 versus O-Veh (Figure 4K). Among these, downregulated genes were predominantly enriched in immune and inflammatory response pathways (Figure 4L,M), indicating that NSPCC1 could reverse immune-senescence phenotypes. It was worth noting that transcriptomic analysis revealed that NSPCC1-treated aged livers exhibited significant changes in their transcriptional profiles, with gene expression patterns that closely resembled those of the young control group (Figure 4N). We also performed KEGG and GO enrichment analyses on the upregulated genes. Enriched pathways between the upregulated genes included fatty acid metabolism, mitochondrial metabolic processes, and the peroxisomal pathway (Figure S3A,B), indicating that NSPCC1 regulated metabolic homeostasis at the transcriptional level. Furthermore, a comparative analysis of metabolism-related DEGs between the O-NSPCC1 and Y-Veh groups revealed that NSPCC1 treatment effectively reversed age-related metabolic changes (Figure S3C).

Collectively, these findings indicated that NSPCC1 administration modulated metabolic balance and inflammatory infiltration, thereby alleviating tissue and organ aging in mice.

### 3.5. NSPCC1 Alleviated Myeloid Differentiation in the Hematopoietic System of Irradiation-Aged Mice

Bone marrow aging was one of the root causes of systemic inflammatory dysregulation. In aging organisms, alterations in the interactions between the bone marrow microenvironment and hematopoietic stem cells caused myeloid progenitor cells to preferentially generate pro-inflammatory monocytes and neutrophils, leading to inflammaging. Jiang et al. found that aged mice exhibited abnormal myelopoiesis following traumatic brain injury, characterized by excessive output of activated neutrophils and classical monocytes from the bone marrow, coupled with a deficiency in non-classical monocytes, which directly led to severe neuroinflammatory infiltration in the brain [31]. Lee et al. further elucidated the regulatory mechanisms in myeloid cells, discovering that upregulation of the FoxO1 gene was a key driver of the progression of non-alcoholic steatohepatitis (NASH). Conditional knockout of FoxO1 in myeloid cells significantly inhibited M1 macrophage polarization and reduced macrophage infiltration in the liver, thereby alleviating tissue inflammation and fibrosis [32]. In summary, these studies provided strong evidence that abnormal expansion or dysfunction of the bone marrow myeloid cell population was a primary driver of tissue inflammatory infiltration in aged mice.

**Figure 5:**
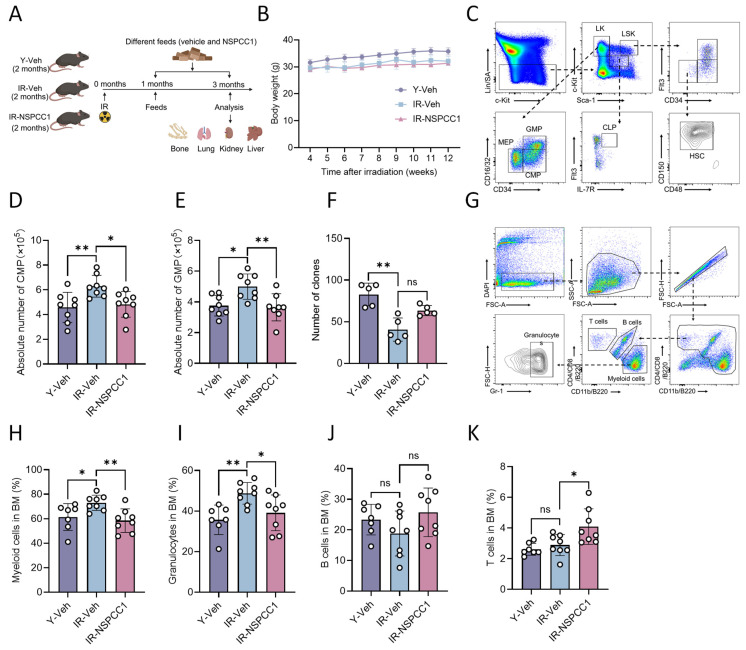
NSPCC1 attenuated irradiation-accelerated myeloid differentiation in the hematopoietic system of irradiation-induced aging mouse model. (**A**) A schematic illustration of the irradiation-induced premature aging model and NSPCC1 intervention. Young mice were subjected to irradiation and then treated with vehicle or NSPCC1. Bone marrow, lung, kidney, and liver tissues were collected for subsequent analyses. (**B**) Longitudinal analysis of body weight in Y-Veh, IR-Veh, and IR-NSPCC1 mice during the feeding period after irradiation. (**C**) Representative flow cytometry gating strategy for bone marrow hematopoietic stem cells and progenitor cells (HSPCs). Gating was performed to detect CMP, GMP, MEP, CLP, and HSC. (**D**,**E**) Absolute numbers of CMPs (**D**) and GMPs (**E**) in bone marrow. (**F**) Quantitative analysis of total colony-forming units (CFUs) after 10 days of in vitro culture, reflecting the clonogenic capacity of bone marrow hematopoietic progenitor cells. (**G**) Representative flow cytometry gating strategy for bone marrow immune cell populations. After excluding dead cells, debris, and doublets, single-cell populations were analyzed. Granulocytes, T cells, B cells, and myeloid cells were identified according to the indicated lineage markers. (**H**–**K**) Quantitative analysis of bone marrow immune cell populations, including myeloid cells (**H**), granulocytes (**I**), B cells (**J**), and T cells (**K**). For body weight and flow cytometry analysis, *n* = 8 mice per group. For colony-forming assays, *n* = 4 biological replicates per group. Data are presented as the mean ± SD. For comparisons between three groups, one-way ANOVA followed by Tukey’s multiple comparisons test was used for normally distributed data. For datasets that did not pass the Shapiro–Wilk normality test, the Kruskal–Wallis test followed by Dunn’s multiple comparisons test was used. Nonparametric analysis was applied to CFU quantification. ns, not significant; * *p* < 0.05, ** *p* < 0.01.

Based on these findings, we hypothesized that NSPCC1 treatment may alleviate tissue and organ aging phenotypes by modulating hematopoietic stem/progenitor cell lineage differentiation in aged mice, thereby influencing downstream immune cell infiltration. To test whether NSPCC1 affected hematopoietic remodeling in irradiation-induced premature aging, 2-month-old mice were exposed to 4.5 Gy of whole-body irradiation and then fed either a vehicle diet or an NSPCC1 diet for 2 months (Figure 5A). NSPCC1 treatment did not significantly affect body weight compared with the IR-Veh group (Figure 5B). Flow cytometric analysis of bone marrow HSPCs showed that irradiation increased the absolute numbers of CMPs and GMPs, whereas NSPCC1 significantly reduced both populations (Figure 5C–E). In contrast, MEPs, LSK cells, CLPs, and HSCs were not significantly altered by NSPCC1 treatment (Figure S4A–D). Colony-forming assays showed that irradiation reduced the total number of colonies formed by bone marrow cells, whereas NSPCC1 treatment partially restored colony-forming capacity, although the increase in total colony number did not reach statistical significance (Figure 5F). Subtype analysis further indicated that this effect was mainly attributable to a significant increase in CFU-GM formation, while BFU-E and CFU-GEMM colonies were not significantly altered (Figure S4E–G). Analysis of mature bone marrow cell populations demonstrated that NSPCC1 reduced the proportion of CD11b^+^ myeloid cells and granulocytes in irradiated mice (Figure 5G–I), whereas B-cell and T-cell proportions were not significantly changed (Figure 5J,K). Collectively, these results suggested that NSPCC1 partially alleviated irradiation-associated myeloid skewing and improved the functional output of bone marrow progenitor cells.

### 3.6. NSPCC1 Alleviated Renal Fibrosis in Irradiation-Aged Mice

**Figure 6:**
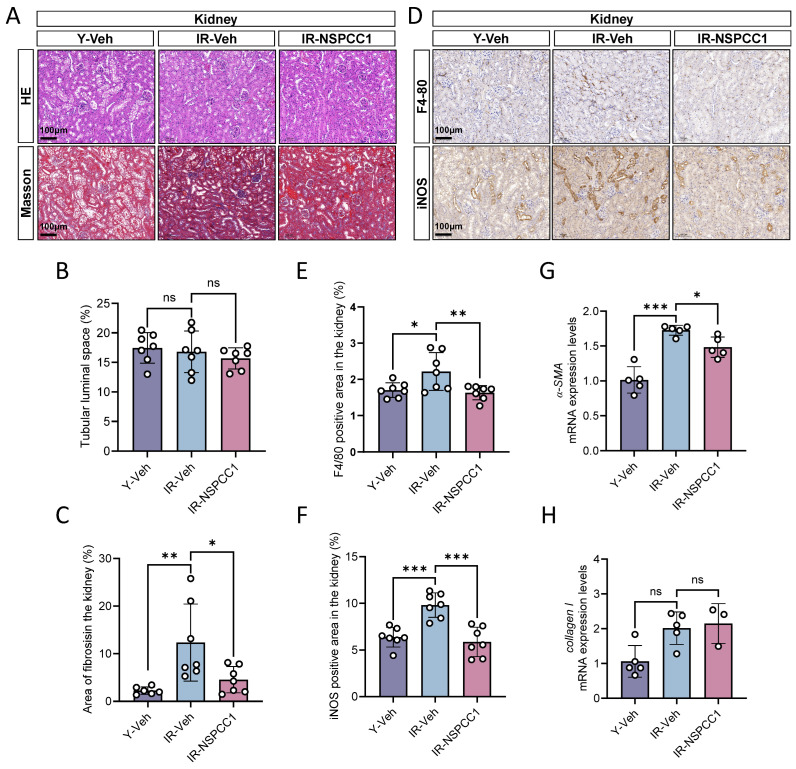
NSPCC1 significantly attenuated renal fibrosis in irradiation-induced aging mouse models. (**A**) Representative H&E and Masson’s trichrome staining images of kidney tissues from Y-Veh, IR-Veh, and IR-NSPCC1 mice. H&E staining was used to evaluate renal histopathological changes, and Masson’s trichrome staining was used to assess collagen deposition. Scale bars: 100 μm. (**B**) Quantitative analysis of the tubular luminal space in the renal cortex. (**C**) Quantitative analysis of the renal fibrotic area. The fibrotic area was quantified as the percentage of Masson-positive collagen area relative to the total analyzed kidney area. (**D**) Representative immunohistochemical staining images of F4/80 and iNOS in kidney tissues from the indicated groups. F4/80 was used as a macrophage marker, and iNOS was used as a marker of pro-inflammatory M1-like macrophages. Scale bars: 100 μm. (**E**) Quantitative analysis of F4/80-positive staining area in kidney tissues. The F4/80-positive area was quantified as the percentage of positive staining area relative to the total analyzed kidney area. (**F**) Quantitative analysis of iNOS-positive staining area in kidney tissues. The iNOS-positive area was quantified as the percentage of the positive staining area relative to the total analyzed kidney area. (**G**,**H**) qPCR analysis of fibrosis-related genes, including α-smooth muscle actin (α-SMA) (**G**) and collagen type I (Collagen 1) (**H**), in kidney tissues. Data are presented as the mean ± SD. Each dot represents one mouse or one biological replicate. *n* = 6–8 mice per group for histological and immunohistochemical analyses; *n* = 4–5 biological replicates per group for qPCR analysis. Histological and immunohistochemical quantification was performed using ImageJ software from representative fields of each kidney section. Differences between the three groups were analyzed using one-way ANOVA followed by Tukey’s multiple comparisons test for normally distributed data. ns, not significant; * *p* < 0.05, ** *p* < 0.01, *** *p* < 0.001.

Using histopathological methods, we evaluated liver, kidney, and lung tissues from aged irradiated mice. H&E staining revealed no statistically significant differences in the number of microgranulomas (Figure S5A,B), tubular lumen space (Figure 6A), or alveolar wall thickness (Figure S5C) following NSPCC1 treatment. We also evaluated the fibrotic changes in liver, kidney, and lung tissues. Masson staining revealed that NSPCC1 treatment significantly ameliorated renal fibrosis (Figure 6A–C), whereas no statistically significant improvements were observed in liver or lung tissues (Figure S5D–F). Our findings indicated that kidney tissue exhibited the most pronounced aging phenotypes in the irradiation-induced aging model, with F4/80 and iNOS staining analyses revealing marked parallel expression. Remarkably, NSPCC1 treatment demonstrated significant efficacy in mitigating these aging-related manifestations (Figure 6D–F).

We further examined macrophage infiltration in liver and lung tissues. F4/80 staining revealed significantly increased macrophage infiltration in irradiated mouse organs, which was effectively attenuated by NSPCC1 treatment (Figure S5G–I). iNOS staining indicated that NSPCC1 similarly reduced radiation-induced increases in M1 macrophage numbers in the lung (Figure S5J,L), but this effect was not observed in the liver (Figure S5J,K).

QPCR analysis showed that α-SMA expression was significantly increased in the kidney of irradiated mice and was reduced after NSPCC1 treatment (Figure 6G), whereas Collagen 1 expression in the kidney showed an increasing trend after irradiation but was not significantly altered by NSPCC1 (Figure 6H). In the liver, neither α-SMA nor Collagen 1 expression differed significantly among groups (Figure S5M,N). In the lung, α-SMA expression was not significantly changed, whereas Collagen 1 expression was significantly increased after irradiation and was reduced by NSPCC1 treatment (Figure S5M,N). These findings suggested that the fibrotic response to irradiation differed across tissues, with the kidney and lung showing greater sensitivity than the liver under the present experimental conditions.

In summary, these data indicated that NSPCC1 markedly attenuated myeloid cell differentiation within the hematopoietic system, thereby reducing immune cell infiltration (particularly M1-polarized macrophages) in tissues and organs. The reduction in macrophage infiltration directly mitigated inflammatory senescence, ultimately extending the organismal lifespan.

## 4. Discussion

Notably, although NSPCC1 improved grip strength in naturally aged mice, its effects on other behavioral endpoints, including locomotor activity, recognition memory, and motor coordination, were limited. This finding suggested that the anti-aging effects of NSPCC1 were not uniform across all functional domains but exhibited a degree of tissue- and function-specific selectivity. Previous studies have shown that the accumulation of senescent cells in vivo exhibits marked tissue specificity. Yousefzadeh et al. systematically compared senescence markers across multiple tissues in naturally aged and progeroid mouse models and found that senescence-associated changes were more pronounced in peripheral tissues such as the liver, kidney, lung, and skin, whereas the senescent cell burden in brain tissue was relatively low [33]. Therefore, the limited improvement in neurobehavioral parameters observed with NSPCC1 may be related to the relatively low accumulation of senescent cells in the brain, as well as to the organ-selective nature of senescent cell clearance interventions.

Nonetheless, this study had several limitations. First, NSPCC1 was a PCC1-enriched composite extract rather than a single purified compound, and its advantages over PCC1 alone or conventional GSE may have reflected not only increased PCC1 content but also the contributions of accompanying components to stability, absorption, and synergistic activity. Because the preparation system involved process protection, the specific contribution of each component could not be fully resolved. Second, although male and female mice were included in relatively balanced numbers, sex-specific analyses were limited in scope. With the exception of survival data, most datasets were analyzed in a pooled manner rather than systematically stratified by sex. Therefore, the potential influence of sex on aging-related phenotypes and immune parameters could not be comprehensively evaluated. Third, the translational relevance of the present findings should be interpreted cautiously, as human aging is highly heterogeneous and may not be directly modeled by the controlled experimental conditions, intervention duration, and pharmacokinetic features observed in mice. In addition, although NSPCC1 appeared to be generally well tolerated under the present conditions, its safety profile was not systematically characterized, and long-term toxicology, off-target effects, maximum tolerated dose, and potential herb–drug interactions remain to be determined.

In addition, the irradiation-induced premature aging model and the naturally aged model represent distinct biological contexts. Irradiation primarily induces an accelerated aging-like state associated with acute DNA damage and hematopoietic stress, whereas natural aging is a progressive process involving chronic inflammation, stem cell exhaustion, metabolic dysregulation, and organ remodeling. Accordingly, findings from irradiated mice should not be considered fully equivalent to those from physiological aging. Moreover, although NSPCC1 treatment was associated with improved HSPC-related parameters, reduced inflammatory cell infiltration, and attenuated organ fibrosis, the causal links between these events were not directly established. Therefore, the current data support attenuation of senescence-associated features rather than definitive proof of senescent cell elimination. Further studies integrating rigorous toxicological evaluation, model-specific validation, and mechanistic interrogation will be required to substantiate NSPCC1’s therapeutic potential.

In recent years, there has been extensive research on natural anti-aging drugs, particularly amid growing attention to traditional medicines and supplements. This research encompassed a wide range of forms, from plant extracts to compound formulations, including resveratrol [34], curcumin [35], astaxanthin [36], green tea extract [37], Liuwei Dihuang [38], and Bazhen decoction [39]. These substances exerted their anti-aging effects through mechanisms such as antioxidant activity, inflammation suppression, and metabolic regulation. Additionally, natural anti-aging supplements, such as NMN [40] and NR [41], gained popularity among consumers for their potential rejuvenating properties. However, challenges remained in the standardization, quality control, and scientific validation of natural products, necessitating more cellular, animal, and even human clinical data.

Despite current limitations in our understanding of NSPCC1’s absorption mechanisms, this study established a foundational approach to addressing aging by remodeling the hematopoietic system. Furthermore, we proposed an integrative four-dimensional evaluation framework that combined HSPC dynamics, peripheral biomarkers, organ fibrosis regression, and lifespan extension. This framework offered a novel standard for assessing natural anti-aging therapeutics and was extensible to mechanistic studies of other natural products. It also informed the development of carrier-enhanced delivery systems, addressing critical challenges in nutraceutical bioavailability.

In conclusion, this work highlighted the potential of hematopoietic-targeted interventions as a transformative frontier in longevity science. Our findings provided a utility for evaluating the efficacy of natural anti-aging compounds using biomarkers that target HSPC dynamics and offered a platform for advancing our understanding of the molecular mechanisms underlying aging across multiple scales. This research laid the possibility for future studies aimed at translating these insights into clinical applications, ultimately contributing to the extension of human lifespan through targeted interventions.

## 5. Conclusions

In this study, we demonstrated that process-optimized NSPCC1 significantly improved the plasma bioavailability of proanthocyanidin C1, an important bioactive compound, at equivalent dosages compared with traditional grape seed extract. Furthermore, we revealed that NSPCC1 influenced systemic aging through a regulatory axis involving hematopoietic stem and progenitor cells. Specifically, it directed the differentiation of hematopoietic stem and progenitor cells, including common myeloid progenitors and granulocyte–monocyte progenitors, thereby enhancing erythropoiesis; increasing antioxidant capacity; and reducing the infiltration of pro-inflammatory M1 macrophages in aging-affected organs, such as the liver, kidneys, and lungs. These synergistic effects contributed to a reduction in tissue fibrosis, alleviated age-related histopathological damage, and promoted an extension of lifespan. Overall, our findings elucidated the “HSPC–circulatory system–multiorgan aging” axis as a critical regulator of organismal aging.

## Data Availability

The data that support the findings of this study are openly available in the NCBI database at https://www.ncbi.nlm.nih.gov/sra/PRJNA1281320 (accessed on 1 August 2025).

## Supplementary Materials

The following supporting information can be downloaded at: https://www.mdpi.com/article/10.3390/biology15120922/s1.

## Abbreviations

PCC1	Proanthocyanidin C1
GSE	Grape seed extract
NSPCC1	Natural Senolytics PCC1
IL-6	Interleukin-6
TNF-α	Tumor Necrosis Factor α
CRP	C-reactive protein
HSCs	Hematopoietic stem cells
DNA	Deoxyribonucleic acid
NF-κB	Nuclear Factor Kappa B
SD rats	Sprague Dawley rats
Y-Veh	Young—Vehicle
O-Veh	Old—Vehicle
O-NSPCC1	Old—Natural Senolytics PCC1
TBI	Total body irradiation
RBC	Red blood cell
HGB	Hemoglobin
HCT	Hematocrit
GSH	Reduced glutathione
GSSG	Oxidized glutathione
T-GSH	Total glutathione
BM	Bone marrow
CD4	Cluster of Differentiation 4
CD8	Cluster of Differentiation 8
B220	B-cell marker 220
CD11b	Cluster of Differentiation 11b
Gr-1	Granulocyte-1
TER119	Ter119 antigen
IL-7R	Interleukin-7 Receptor
FLT3	Fms-like Tyrosine Kinase 3
CD150	Cluster of Differentiation 150
CD48	Cluster of Differentiation 48
Sca-1	Stem Cell Antigen-1
c-Kit	KIT proto-oncogene receptor tyrosine kinase
CD16/32	Cluster of Differentiation 16/32
CD34	Cluster of Differentiation 34
CFU	Colony-Forming Unit
BFU-E	Burst-Forming Unit—Erythroid
CFU-GM	Colony-Forming Unit—Granulocyte/Macrophage
CFU-GEMM	Colony-Forming Unit—Granulocyte/Erythrocyte/Macrophage/Megakaryocyte
H&E	Hematoxylin and eosin staining
BSA	Bovine serum albumin
DAB	Diaminobenzidine
LC-MS	Liquid chromatography–mass spectrometry
AUC	Area under the curve
Cmax	Maximum plasma concentration
Tmax	The time to maximum concentration
HSPCs	Hematopoietic stem and progenitor cells
qPCR	Quantitative polymerase chain reaction
CCL2/CCR2	C-C Motif Chemokine Ligand 2/C-C Motif Chemokine Receptor 2
IL-1β	Interleukin-1β
TGF-β/Smad3	Transforming Growth Factor-beta/Smad3
F4/80	EGF-like Module-containing Mucin-like Hormone Receptor-like 1
iNOS	Inducible nitric oxide synthase
PCA	Principal component analysis
DEGs	Differentially expressed genes
KEGG	Kyoto Encyclopedia of Genes and Genomes
GO	Gene Ontology
MEPs	Megakaryocyte–erythroid progenitors
LSKs	Lineage-Sca-1+c-Kit+ cells
CLPs	Common lymphoid progenitors
