# Supplementary material for: Hematopoietic Rejuvenation via Natural Senolytic NSPCC1 Delays Inflammatory Aging
Source: Biology (Basel). 2026 Jun 12;15(12):922. doi: 10.3390/biology15120922 (PMC13296190; doi:10.3390/biology15120922)
Supplement: Supplementary file 1 [file biology-15-00922-s001.zip › Supplementary information.pdf]

SUPPLEMENTARY INFORMATION

Hematopoietic rejuvenation by natural senolytic NSPCC1 delays inflammatory aging

Wei Wang[1,2], Shenglong Yang[1,2], Rongjinlei Zhang[1,2], Yufang Wang[3], Zhen Zhang[3], Feng Xiao[2], Shu Wu[1,2], Zhenyu Ju[1,2], Ruikun He[3*], Yuanlong Ge[1,2*]

[1]State Key Laboratory of Bioactive Molecules and Druggability Assessment, Guangdong Basic Research Center of Excellence for Natural Bioactive Molecules and Discovery of Innovative Drugs, College of Life Science and Technology, Jinan University, Guangzhou 510632, Guangdong, China.

[2]Key Laboratory of Regenerative Medicine of Ministry of Education, Institute of Aging and Regenerative Medicine, Department of Developmental & Regenerative Medicine, College of Life Science and Technology, Jinan University, Guangzhou 510632, Guangdong, China.

[3]BYHEALTH Institute of Nutrition & Health, Guangzhou, 510663, China.

To whom correspondence should be addressed, E-mail: geyuanlong@jnu.edu.cn;

Correspondence may also be addressed to:

Ruikun He, E-mail: herk@by-health.com.

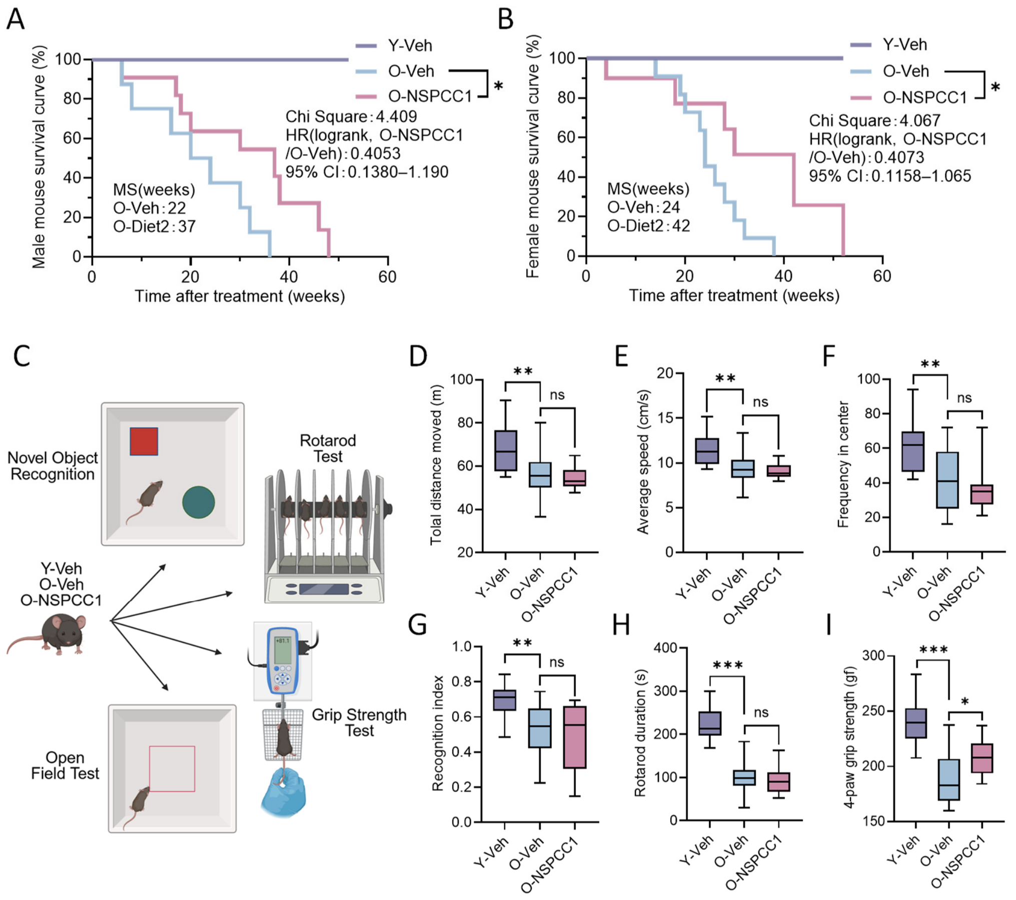

Supplementary Figure S1. Sex-stratified survival analysis and behavioral assessment of naturally aged mice treated with NSPCC1.

(A,B) Kaplan–Meier survival curves of male mice (A) and female mice (B) in the O-Veh, and O-NSPCC1 groups during the treatment period. The median survival time of mice in the O-Veh and O-NSPCC1 groups was calculated, and the log-rank Mantel-Cox test was used to analyze survival differences. (C) A schematic illustration of behavioral assessments performed in naturally aged mice, including the open field test, novel object recognition test, rotarod test, and grip strength test. (D–F) Quantitative analysis of open field test parameters, including total distance moved (D), average speed (E), and frequency in the center area (F), reflecting locomotor

activity and anxiety-like behavior. (G) Quantitative analysis of the recognition index in the novel object recognition test, reflecting recognition memory. (H) Quantitative analysis of latency to fall in the rotarod test, reflecting motor coordination and balance. (I) Quantitative analysis of four-limb grip strength, reflecting muscule function. For behavioral analyses, sample sizes were as follows: Y-Veh, n = 10; O-Veh, n = 12; and O-NSPCC1, n = 13. Data are presented as box-and-whisker plots showing the median, interquartile range, and minimum-to-maximum values. Survival curves were analyzed using Kaplan–Meier survival analysis followed by the log-rank Mantel–Cox test. For behavioral comparisons among three groups, one-way ANOVA followed by Tukey's multiple comparisons test was used for normally distributed data. ns, not significant; *$P < 0.05$, **$P < 0.01$, ***$P < 0.001$.

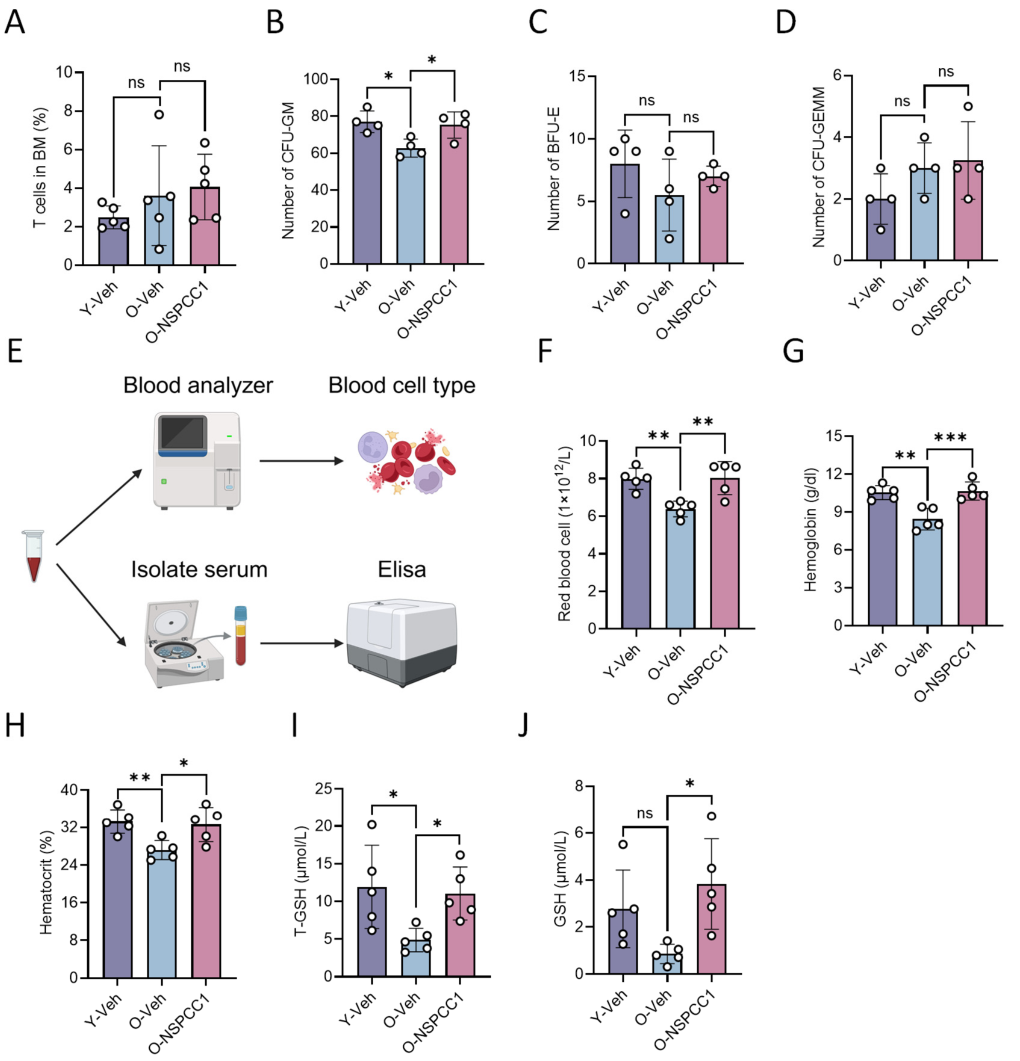

Supplementary Figure S2. NSPCC1 ameliorated hematopoietic aging in naturally aged mice.

(A) The quantitative analysis of bone marrow T cell populations in the Y-Veh, O-Veh, and O-NSPCC1 groups. T cells were analyzed based on $CD4^{+}$ and $CD8^{+}$ lymphocyte populations. (B–D) Colony-forming assays were performed to evaluate the clonogenic capacity of bone marrow hematopoietic progenitor cells after 10 days of in vitro culture. Quantitative analyses included CFU-GM colonies (B), BFU-E colonies (C), and CFU-GEMM colonies (D). (E) A schematic illustration of peripheral blood and

serum analyses. Peripheral blood was collected for hematological analysis using an automated blood analyzer, and serum was isolated for ELISA-based measurement of glutathione-related parameters. (F–H) The quantitative analysis of peripheral blood parameters, including red blood cell count (F), hemoglobin concentration (G), and hematocrit (H). (I,J) The quantitative analysis of serum total glutathione (T-GSH) (I) and reduced glutathione (GSH) (J) concentrations. For bone marrow T cell analysis, n = 5 mice per group. For colony-forming assays, n = 4 biological replicates per group. Data were presented as mean ± SD. Differences among the three groups were analyzed using one-way ANOVA followed by Tukey's multiple comparisons test for normally distributed data. ns, not significant; *P < 0.05, **P < 0.01, ***P < 0.001.

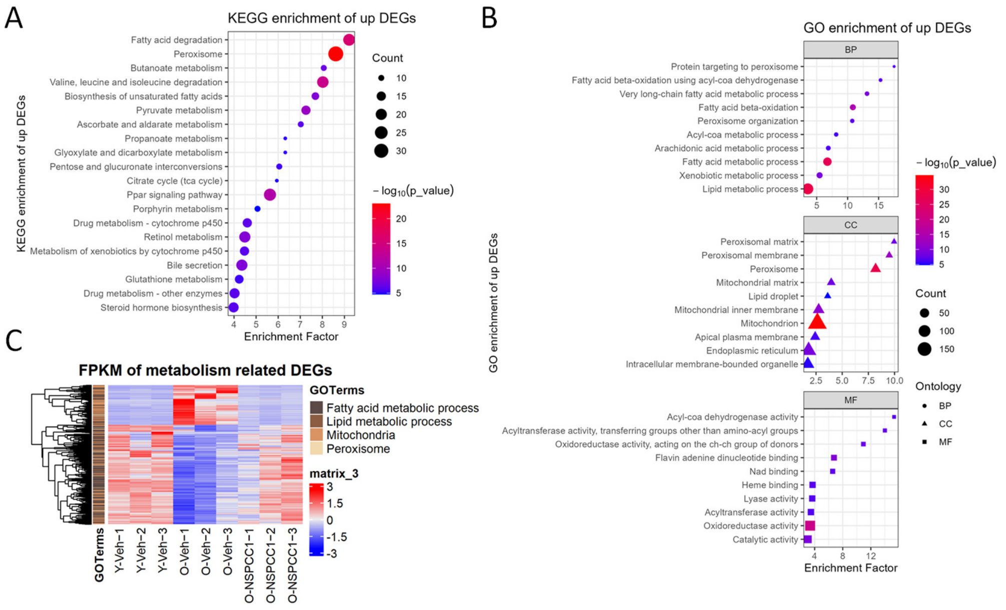

Supplementary Figure S3. NSPCC1 partially restored metabolism-related transcriptional changes in naturally aged mice.

(A–C) Functional enrichment analysis of upregulated differentially expressed genes (DEGs) in the O-NSPCC1 group compared with the O-Veh group. (A) KEGG pathway enrichment analysis of upregulated DEGs. The top enriched pathways were shown according to enrichment significance. (B) Gene Ontology (GO) enrichment analysis of upregulated DEGs, including biological process (BP), cellular component (CC), and molecular function (MF) categories. (C) Heatmap showing the expression patterns of DEGs associated with enriched metabolism-related terms among Y-Veh, O-Veh, and O-NSPCC1 liver samples. The heatmap was generated based on normalized FPKM values. For transcriptomic analysis, n = 3 biologically independent liver samples per group. DEGs were identified using the criteria of |log2FC| > 1 and Q value < 0.05. Enrichment analysis was performed based on upregulated DEGs in the O-NSPCC1 versus O-Veh comparison.

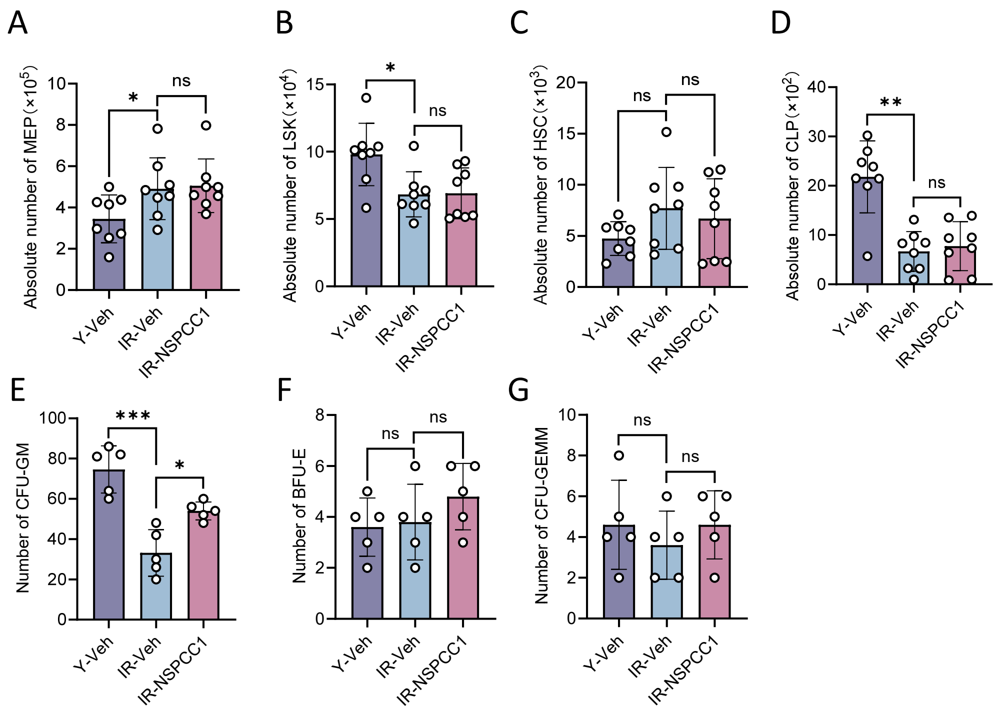

Supplementary Figure S4. NSPCC1 partially improved hematopoietic progenitor function in irradiation-induced premature aging mice.

(A–D) The quantitative analysis of hematopoietic stem and progenitor cell populations in bone marrow from Y-Veh, IR-Veh, and IR-NSPCC1 mice, including MEPs (A), LSK cells (B), HSCs (C), and CLPs (D). (E–G) Colony-forming assays were performed to evaluate the clonogenic capacity of bone marrow hematopoietic progenitor cells after 10 days of in vitro culture. The quantitative analyses included CFU-GM colonies (E), BFU-E colonies (F), and CFU-GEMM colonies (G). For flow cytometry analysis, n = 8 mice per group. For colony-forming assays, n = 5 biological replicates per group. Data were presented as mean ± SD. Differences among the three groups were analyzed using one-way ANOVA followed by Tukey's multiple comparisons test for normally distributed data. ns, not significant; *P < 0.05, **P < 0.01, ***P < 0.001.

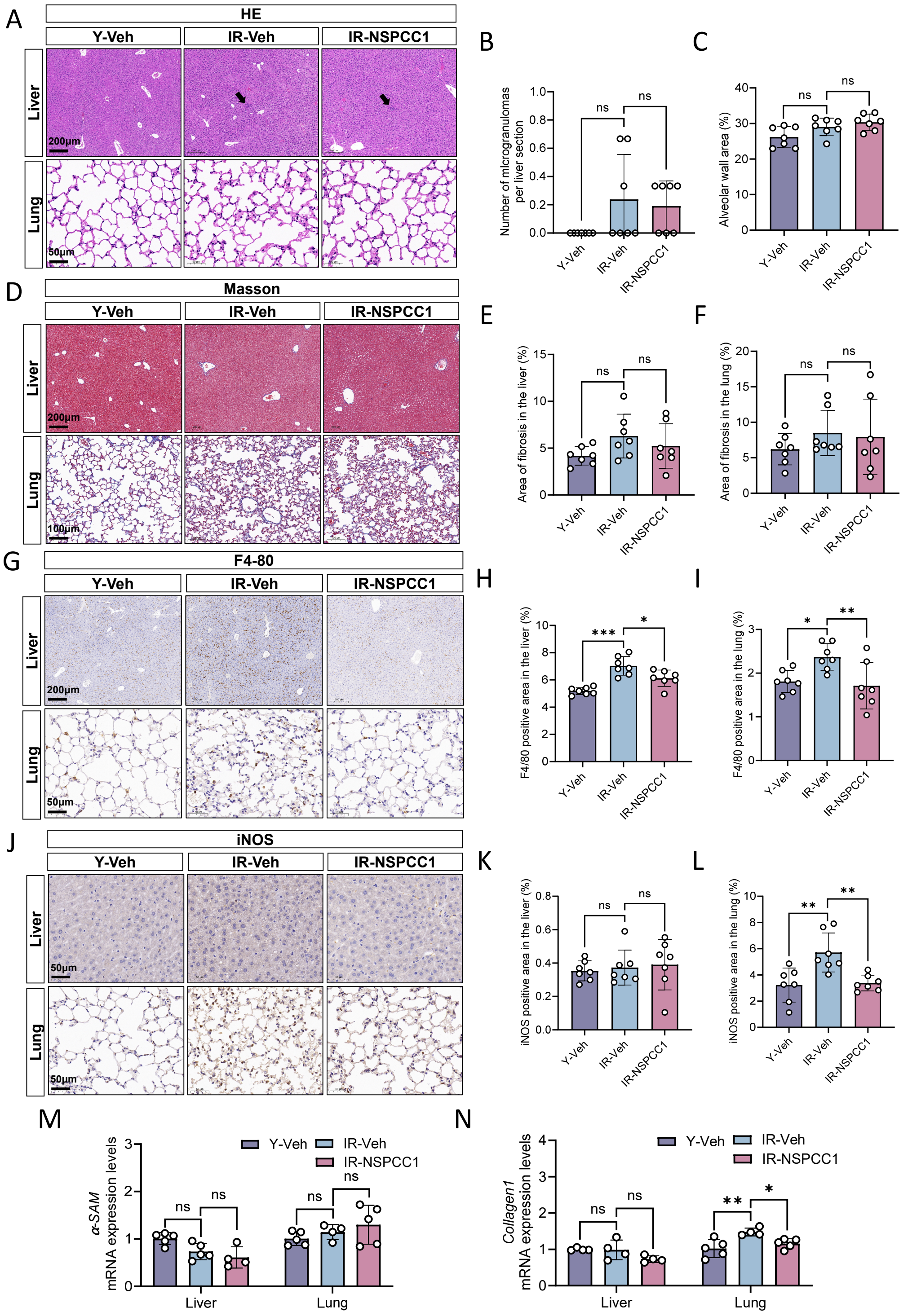

Supplementary Figure S5. NSPCC1 showed limited effects on liver and lung fibrosis but reduced inflammatory infiltration in irradiation-induced premature aging mice.

(A) Representative H&E-stained images of liver and lung tissues from Y-Veh, IR-Veh, and IR-NSPCC1 mice. Arrows indicated hepatic microgranulomas. Scale bars: 200 μm for the liver and 50 μm for the lung. (B,C) The quantitative histopathological analysis of liver and lung tissues, including the number of hepatic microgranulomas per tissue area (B) and the percentage of damaged area in lung tissue (C). (D) Representative Masson's trichrome staining images of liver and lung tissues from the indicated groups. Collagen deposition was shown in blue. Scale bars: 200 μm for the liver and 100 μm for the lung. (E,F) The quantitative analysis of fibrotic area in liver (E) and lung (F). Fibrotic area was quantified as the percentage of Masson-positive collagen area relative to the total analyzed tissue area. (G) Representative immunohistochemical staining images of F4/80 in liver and lung tissues. F4/80 was used as a macrophage marker. Scale bars: 200 μm for liver and 50 μm for lung. (H,I) The quantitative analysis of F4/80-positive staining area in liver (H) and lung (I). F4/80-positive area was quantified as the percentage of positive staining area relative to the total analyzed tissue area. (J) Representative immunohistochemical staining images of iNOS in liver and lung tissues. iNOS was used as a marker of pro-inflammatory M1-like macrophages. Scale bars: 50 μm for liver and 50 μm for lung. (K,L) The quantitative analysis of iNOS-positive staining area in liver (K) and lung (L). iNOS-positive area was quantified as the percentage of positive staining area relative to the total analyzed tissue area. (M,N) qPCR analysis of fibrosis-related genes, includingα-SMA (M) and Collagen 1 (N), in liver and lung tissues. Data were presented as mean ± SD. Each dot represented one mouse or one biological replicate.

Histological and immunohistochemical quantification was performed using ImageJ software based on representative fields from each tissue section. Differences among the three groups were analyzed using one-way ANOVA followed by Tukey's multiple comparisons test for normally distributed data. For datasets that did not pass the Shapiro–Wilk normality test, the Kruskal–Wallis test followed by Dunn's multiple comparisons test was used. Nonparametric analysis was applied to lung fibrotic area and liver iNOS-positive area. ns, not significant; *$P < 0.05$, **$P < 0.01$, ***$P < 0.001$.
